# Histological Changes in Adrenal Glands in Suicidal and Sudden Death Cases: A Postmortem Study

**DOI:** 10.7759/cureus.54630

**Published:** 2024-02-21

**Authors:** Sangita Chaurasia, Anshuli Trivedi, Ruchi Ganvir, Saagar Singh, Jayanthi Yadav, Reeni Malik, Sneha Choubal, Arneet Arora

**Affiliations:** 1 Department of Forensic Medicine and Toxicology, Gandhi Medical College, Bhopal, IND; 2 Department of Community Medicine, Gandhi Medical College, Bhopal, IND; 3 Department of Microbiology, L. N. Medical College and Research Centre, Bhopal, IND; 4 Department of Forensic Medicine and Toxicology, Dr. Laxmi Narayan Pandey Medical College, Ratlam, IND; 5 Department of Forensic Medicine and Toxicology, All India Institute of Medical Sciences, Bhopal, Bhopal, IND; 6 Department of Pathology, Gandhi Medical College, Bhopal, IND; 7 Department of Pathology, Parakh Pathology Lab, Bhopal, IND

**Keywords:** chronic stress, acute stress, suicide, histological changes, adrenal gland

## Abstract

Introduction

Various studies have linked suicidal behavior, stress, affective disorders, and dysregulation of the hypothalamic-pituitary-adrenal (HPA) axis resulting from chronic stress. Chronic stress has been shown to cause enlargement of the adrenal glands, altering their function and potentially leading to suicidal behaviors in individuals with depression. This study aimed to compare the histological changes in the adrenal glands of individuals who died by suicide with those who experienced sudden death. Suicide victims are exposed to chronic stress, while individuals with sudden deaths face acute stress related to the act of dying.

Methods

This analytical study, approved by the Institutional Ethics Committee (IEC), was conducted in the Department of Forensic Medicine and Toxicology at Gandhi Medical College, Bhopal. The study included 100 confirmed cases of suicide, irrespective of gender, aged 15-60 years, with notable autopsy findings, relevant history, no signs of decomposition, and varying survival periods (including immediate deaths within 24 hours). Additionally, 20 controls were selected, involving individuals who died suddenly from causes other than suicide within 24 hours of the incident. Informed consent was obtained using a prescribed proforma from relatives and the police. Histological examination slides of the adrenals were prepared and analyzed. Data were collected and statistically analyzed using GraphPad software and Epi Info 7.

Results

Capsular hemorrhage was observed in 98% of suicide cases and 40% of controls. Nodulation was present in 48% of suicidal cases and 20% of controls. Zonal extension of zona fasciculata was specific to chronic stress in suicidal cases. In 25% of suicidal cases, a prominent extension of the medulla was noted. Irregular thinning of zona glomerulosa with shrunken cells and increased nuclear density in 88% of cases were considered specific to chronic stress conditions and suicide, not observed in controls. Lipid depletion was observed in all suicidal cases, with diffuse depletion in 47% and focal depletion in 53% of cases. In contrast, 45% of those exposed to the acute stress of dying showed focal depletion, with none exhibiting diffuse depletion. Suicidal cases displayed dilated prominent sinusoids in all three zones and the adrenal medulla (98-99%), absent in controls. Adrenal hemorrhage and necrosis were specific to chronic stress conditions, with 7%, 8%, 32%, and 16% of cases showing hemorrhage in all three zones and adrenal medulla, respectively, and none in controls.

Conclusion

Histological changes observed in acute stress conditions included focal lipid depletion, capsular hemorrhage, nodular hyperplasia, and hemorrhage and necrosis with edema. However, the proportion and severity of these changes were lower than those observed in the suicidal group, suggesting that these findings may be considered non-specific for differentiating between acute and chronic stress.

## Introduction

Each year, approximately 700,000 individuals take their own lives, making suicide the fourth most prevalent cause of death among those aged 15-29. A staggering 77% of all suicides occur in low- and middle-income countries worldwide [1]. In India, suicide has emerged as a significant and escalating public health issue. In 2016, the suicide mortality rate was 16.5 per 100,000 population, surpassing the global average of 10.5 per 100,000, and increased to 18.3 according to the latest data [2].

According to the WHO, suicide is defined as 'deliberate harm to oneself and self-injury with varying degrees of lethal intent and fatal outcome' [3]. Suicidal behavior is closely associated with diagnosable psychiatric disorders and can be triggered by both acute and chronic stress [4]. Under stress, the anterior pituitary releases adrenocorticotropic hormone (ACTH), which reaches the adrenal gland cortex, stimulating the secretion of cortisol, the primary stress hormone from endocrine cells. This physiological stress response, known as the general adaptation syndrome, is facilitated by activating the hypothalamic-pituitary-adrenal (HPA) axis [5].

However, chronic stress disrupts the normal homeostasis of the HPA axis, leading to chronic fatigue of the adrenal gland and resulting in hypertrophy and hyperplasia of the adrenal glands. Various research studies have established connections between suicidal conduct, stress, affective disorders, and dysregulation of the HPA axis [5,6]. Subsequently, elevated total plasma cortisol has been proposed as a predictor for suicide [6]. These findings suggest that chronic stress induces enlargement of the adrenal glands, altering their function and potentially contributing to suicidal behaviors in individuals experiencing depression.

Limited studies have investigated the histological changes in the adrenal glands of depressed individuals who have committed suicide compared to those who have experienced sudden death. This study aims to compare the distinct histological changes in the adrenal glands of individuals who died by suicide, exposed to chronic stress, and those who experienced sudden death and were subjected to the acute stress of dying.

## Materials and methods

Study design and participants

This analytical and comparative observational study was undertaken at the Department of Forensic Medicine and Toxicology of Gandhi Medical College, Bhopal. The reporting and article preparation for the cross-sectional aspects of the study adhered to the Strengthening the Reporting of Observational Studies in Epidemiology (STROBE) recommendations. The present study entitled 'Histological Changes in Adrenal Glands in Suicidal and Sudden Death Cases: A Postmortem Study' was approved by the Institutional Ethics Committee of Gandhi Medical College, Bhopal, India, with approval number 17219-22/MC/7/2017.

Inclusion and exclusion criteria

The study comprised 100 confirmed cases of suicide, spanning both genders and ages 15-60, with distinct autopsy findings, relevant history, and no signs of decomposition. Cases with a survival period ranging from immediate death to within 24 hours were included. Efforts were made to exclude cases with insufficient or doubtful history, hospitalization for more than 24 hours, age under 15 or over 60 years, chronic gross pathology in any organ, ingestion of alcohol at autopsy, known cases of depression with antidepressant treatment, pregnant individuals, and mutilated bodies. Additionally, 20 controls were selected, including those who died suddenly from non-suicidal causes such as electrocution, road traffic accidents, acute cardiac causes without gross pathology, or homicidal deaths from blunt, stab, or firearm injuries occurring within 24 hours of the incident. Consent was obtained from relatives and the police before starting the study.

Data sources and variables

Height and weight measurements were recorded according to guidelines. External and internal body examinations were conducted to exclude gross pathology, including a meticulous examination of all visceral organs.

Tissue sampling and processing

Kidneys, peri-renal fat, and adrenal glands on both sides were carefully removed and separated without tearing. The adrenal glands were fixed in 10% formalin for 24 hours. Multiple longitudinal cuts (0.5 cm thick) were made, and tissue samples were preserved in separate labeled bottles. Tissue processing involved initial fixation and re-fixation using 10% formalin, dehydration using ethanol and absolute alcohol, clearing with xylene, infiltration, and impregnation with molten paraffin wax, embedding with paraffin wax, and section cutting (5-micron thickness). Slides were prepared using the H&E staining method.

Microscopic examination

**Figure 1:**
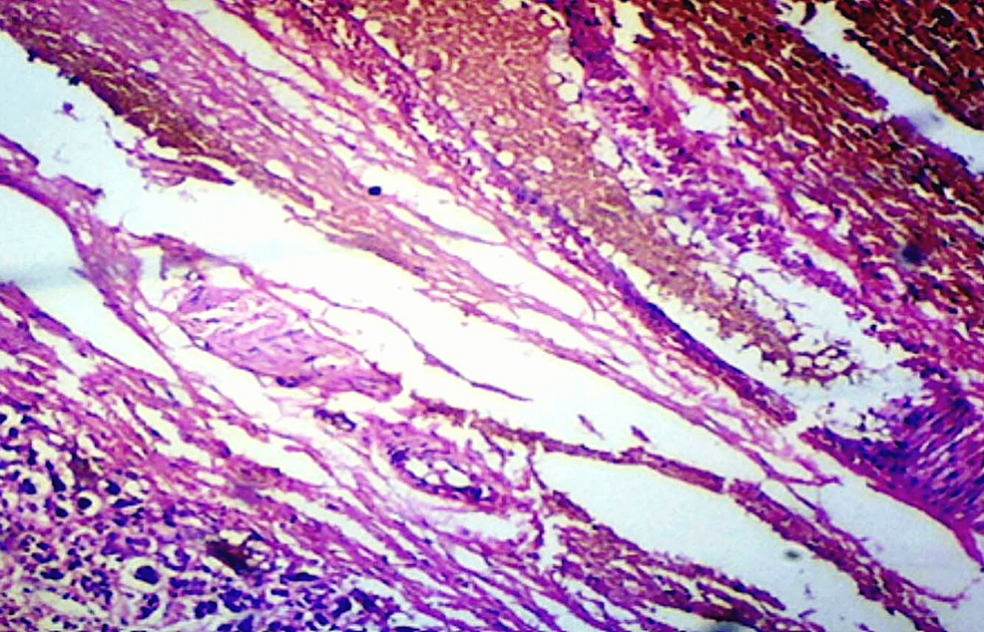
Photomicrograph showing capsular hemorrhage in the adrenal gland, magnified 100x (H&E stain).

The microscopic examination assessed histological changes in the adrenal glands obtained from the studied cases. Slides were meticulously examined under an optical microscope, considering key parameters such as the age and gender of the deceased, the cause of death, and any history of previous suicidal attempts. Various histological changes were observed and categorized under specific headings, each shedding light on distinct aspects of adrenal gland morphology. Capsular hemorrhage, illustrated in Figure 1, was scrutinized to determine the presence and extent of hemorrhage surrounding the adrenal glands.

**Figure 2:**
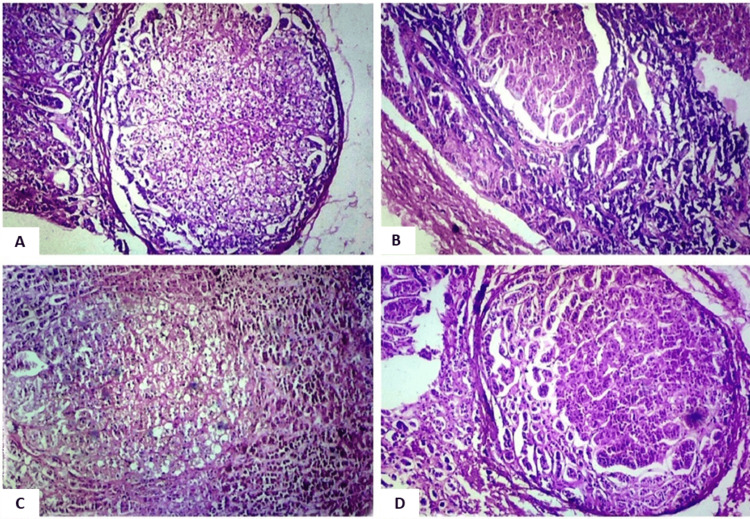
Photomicrograph showing nodule formation (regions of importance have been focused on in the field). A. Nodule external to the capsule, magnified 40x (H&E stain). B. Nodular formation in the zona glomerulosa, magnified 40x (H&E stain). C. Nodular formation in the zona fasciculata, magnified 40x (H&E stain). D. Nodular formation in the zona reticularis with brown lipofuscin pigments in cells, magnified 40x (H&E stain).

Nodular hyperplasia, depicted in Figure 2, was another focal point of the microscopic examination. This aspect aimed to identify and characterize abnormal nodular growths within the adrenal tissue, providing insights into potential pathological developments.

**Figure 3:**
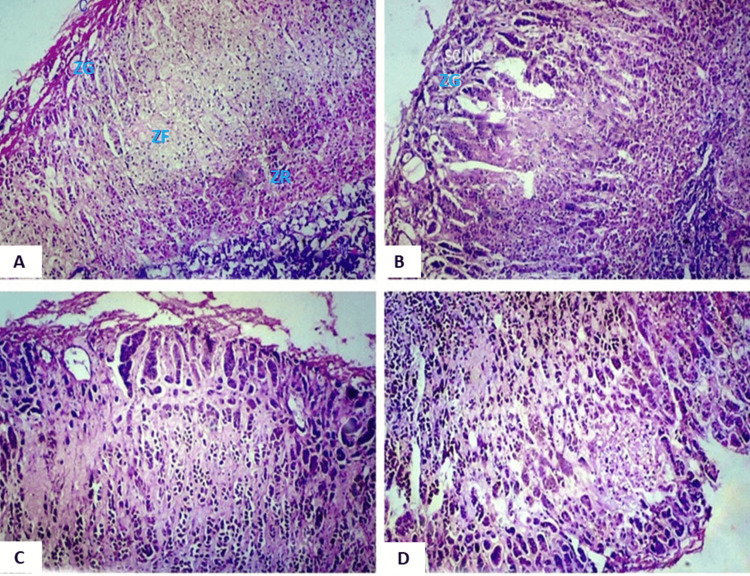
Photomicrograph showing histology of different layers of the adrenal gland (regions of importance have been focused on in the view). A. Histology of the adrenal gland showing normal zonation of the zona reticularis (ZR) and a reticular arrangement of compact cells, magnified 40x (H&E stain). B. Extension of the zona fasciculata (ZF) with thinning of the zona glomerulosa (ZG), magnified 40x (H&E stain). C. Extension of the zona reticularis (ZR) (showing brownish lipofuscin pigments) into the zona fasciculata, magnified 40x (H&E stain). D. Extension of the reticularis into the medulla, magnified 40x (H&E stain).

Intracytoplasmic lipid depletion was examined in detail, with a nuanced approach differentiating between focal and diffuse lipid depletion, as illustrated in Figure 3. This classification aimed to assess the distribution and severity of lipid depletion within the adrenal glands.

**Figure 4:**
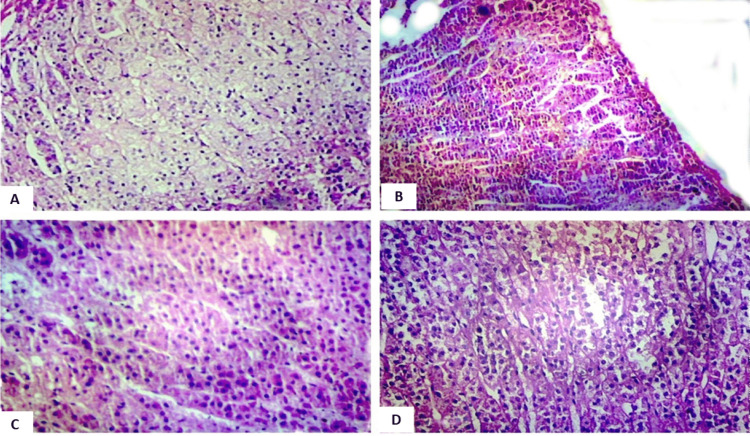
Photomicrograph showing the arrangement of cells (region of importance has been focused on in the view). A. Normal lipid in the zona fasciculata, magnified 40x (H&E stain). B. Focal degree of intracytoplasmic lipid depletion in the zona fasciculata, magnified 40x (H&E stain). C. Diffuse degree of intracytoplasmic lipid depletion in the zona fasciculata with parenchymal cord-like arrangement of cells, magnified 100x (H&E stain). D. Hyperchromatic nuclei in the zona fasciculata, magnified 100x (H&E stain).

Furthermore, attention was given to the parenchymal cord-like arrangement of cells, categorized into focal and diffuse changes, as shown in Figure 4. This allowed for the evaluation of alterations in the arrangement of cells within the adrenal parenchyma.

**Figure 5:**
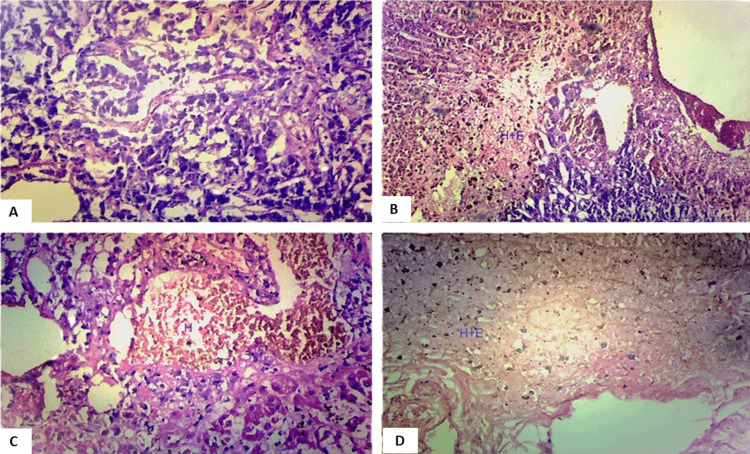
Photomicrograph showing lesions in the adrenal gland (regions of importance have been focused on in the view). A. Sinusoidal dilatation in the medulla, magnified 100x (H&E stain). B. Hemorrhage along with hemosiderin-like pigment and edema in the zona reticularis, magnified 100x (H&E stain). C. Hemorrhage in the medulla with hemosiderin pigments and edema, magnified 100x (H&E stain). D. Hemorrhage in the zona glomerulosa, magnified 100x (H&E stain).

The microscopic examination also explored histological changes in specific zones of the adrenal glands, namely the zona glomerulosa (ZG), zona fasciculata (ZF), and zona reticularis (ZR). Each zone was scrutinized individually to identify and document any discernible tissue structure or cellular composition alterations (Figure 5).

Additionally, the medulla of the adrenal glands underwent thorough microscopic evaluation, examining the cellular architecture and histological changes within the medullary region. The entire process adhered to a systematic approach, documenting observations systematically on slides.

Data analysis

The thorough examination of histological changes in the adrenal glands was followed by meticulous data analysis using advanced statistical tools, namely GraphPad software and Epi Info 7. The collected data were presented as frequencies and percentages, which were then tested using Pearson's chi-square and Fischer's exact tests. Graphs were generated using Microsoft Excel and Microsoft Word. A p-value of <0.05 was considered statistically significant.

This analytical phase aimed to unravel significant patterns and correlations within the collected data, probing the intricate relationship between observed histopathological alterations and various demographic or circumstantial factors. Demographic parameters such as age, gender, cause of death, and history of previous suicidal attempts were scrutinized in conjunction with the identified histological changes. The statistical techniques sought to quantify the prevalence and significance of each observed alteration, offering insights into potential associations with specific circumstances surrounding deaths. This analytical endeavor played a pivotal role in discerning whether particular histopathological features exhibited variations between suicides, exposure to chronic stress, and sudden deaths subjected to acute stress.

## Results

A total of 100 study cases were incorporated into the analysis, consisting of 69 males and 31 females aged 15 to 60 years. Among these cases, 17 fell within the age group of 15 to 20 years, 50 between 21 and 30, 19 between 31 and 40, 7 between 41 and 50, and 7 between 51 and 60. The mean age ± SD for the study group was 29.5 ± 11.4 years. In the control group, there were 16 males and 4 females, with a mean age ± SD of 33.3 ± 11.5 years (Table 1).

**Table 1:** Age and gender-wise distribution of study cases.

Age group (in years)	Male		Female		Total	
	Control	Study	Control	Study	Control	Study
15-20	2	7	0	10	02	17
21-30	7	36	3	14	10	50
31-40	3	16	0	3	3	19
41-50	3	5	1	2	4	7
51-60	1	5	0	2	1	7
Total	16	69	04	31	20	100

**Figure 6:**
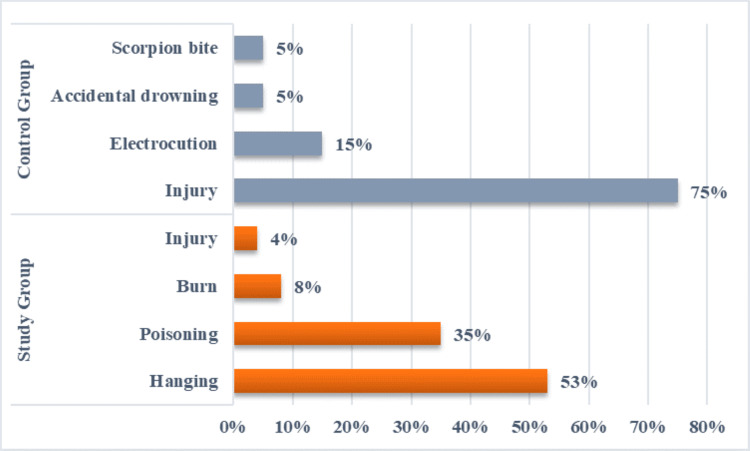
Distribution of cases and controls on the basis of cause of death.

Within the study group, diverse methods of suicide were employed, with hanging being the most prevalent at 53% (N=53), followed by poisoning at 35% (N=35), burn incidents at 8% (N=8), and injuries accounting for 4% (N=4). In contrast, the control group exhibited distinct causes of death, primarily attributed to injuries, including head injuries or multiple injuries resulting from road traffic accidents, homicidal stabs, and cutthroat injuries, constituting 75% (N=15) of the cases. Other causes included electrocution at 15% (N=3), accidental drowning at 5% (N=1), and scorpion bite at 5% (N=1) (Figure 6).

The examination revealed several histological changes in the adrenal glands. Capsular hemorrhage was observed (Figure 1), along with nodular hyperplasia (Figure 2) and alterations in the zona glomerulosa. These changes included variations in cell size, thinning of the zona glomerulosa with shrunken cells and increased nuclear density (Figures 3B, 3D), nuclear prominence, sinusoidal dilation, and hemorrhage (Figure 5D), along with necrosis and edema. Further changes were noted in the zona fasciculata and zona reticularis, encompassing extension (Figures 3B, 3D), alterations in cell size, nuclear prominence (Figure 4D), sinusoidal dilation, and focal and diffuse lipid depletion (Figures 4B, 4C). Additionally, focal and diffuse parenchymal cord-like arrangements of cells were observed (Figure 4C), along with hemorrhage and hemosiderin-like pigments (Figure 5B), accompanied by necrosis and edema.

In the medulla, changes involved extension, alterations in cell size, nuclear prominence, sinusoidal dilation (Figure 5A), hemorrhage (Figure 5C), and necrosis and edema. Capsular hemorrhage was observed in both groups, suicidal deaths with chronic stress and acute stress of dying. Suicidal cases exhibited a significantly higher incidence of capsular hemorrhage (98%) compared to control cases (35-40%), as determined by the two-tailed Fisher’s exact test (p<0.0001) (Table 2).

**Table 2:** Comparison of various histological parameters in the study and control groups.

Parameters		Groups				P-value
		Study group		Control group		
		100 Cases	200 Adrenals	20 Cases	40 Adrenals	
Capsular Haemorrhage	Present	98 (98%)	196 (98%)	8 (40%)	13 (32.5%)	<0.0001
	Absent	2 (2%)	4 (2%)	12 (60%)	27 (67.5%)	
Nodular Hyperplasia	Present	48 (48%)	90 (45%)	4 (20%)	8 (20%)	0.026
	Absent	52 (52%)	110 (55%)	16 (80%)	32 (80%)	
Histological Changes in Zona Glomerulosa	Cell Size	Normal	Normal	Normal	-	-
	Zonal thinning with shrunken cells and increased nuclear density	88 (88%)	176 (88%)	0 (0%)	-	
	Nuclear prominence	68 (68%)	135 (67.5%)	0 (0%)	-	
	Sinusoidal dilatation	98 (98%)	195 (97.5%)	0 (0%)	-	
	Lipid depletion	Not marked	Not marked	Not marked	-	
	Parenchymal cord-like arrangement of cells	Not marked	Not marked	Not marked	-	
	Hemorrhage	7 (7%)	14 (7%)	0 (0%)	-	
	Hemorrhage + Hemosiderin-like pigments	1 (1%)	2 (1%)	0 (0%)	-	
	Edema	4 (4%)	8 (4%)	0 (0%)	-	
	Necrosis	2 (2%)	4 (2%)	0 (0%)	-	
	Edema + Necrosis	2 (2%)	4 (2%)	0 (0%)	-	
Histological Changes in Zona Fasciculata	Extension of Zona Fasciculata	96 (96%)	192 (96%)	0 (0%)	0 (0%)	-
	Cell size	Normal	Normal	Normal	Normal	
	Nuclear prominence	95 (95%)	189 (94.5%)	0 (0%)	0 (0%)	
	Sinusoidal dilatation	99 (99%)	197 (98.5%)	0 (0%)	0 (0%)	
	Focal lipid depletion	53 (53%)	102 (51%)	9 (45%)	17 (42.5%)	
	Diffuse lipid depletion	47 (47%)	94 (47%)	0 (0%)	0 (0%)	
	Focal parenchymal cord-like arrangement of cells	53 (53%)	102 (51%)	0 (0%)	0 (0%)	
	Diffuse parenchymal cord-like arrangement of cells	46 (46%)	92 (46%)	0 (0%)	0 (0%)	
	Hemorrhage	8 (8%)	14 (7%)	0 (0%)	0 (0%)	
	Hemorrhage + Hemosiderin-like pigments	8 (8%)	16 (8%)	0 (0%)	0 (0%)	
	Edema	7 (7%)	14 (7%)	1 (5%)	2 (5%)	
	Necrosis	1 (1%)	2 (1%)	0 (0%)	0 (0%)	
	Edema + Necrosis	3 (3%)	6 (3%)	0 (0%)	0 (0%)	
Histological Changes in Zona Reticularis	Extension of Zona Fasciculata	92 (92%)	182 (91%)	0 (0%)	0 (0%)	-
	Cell size	Normal	Normal	Normal	Normal	
	Nuclear prominence	95 (95%)	189 (92.5%)	0 (0%)	0 (0%)	
	Sinusoidal dilatation	98 (98%)	194 (97%)	0 (0%)	0 (0%)	
	Focal lipid depletion	83 (83%)	161 (80.5%)	4 (20%)	7 (17.5%)	
	Diffuse lipid depletion	13 (13%)	25 (12.5%)	0 (0%)	0 (0%)	
	Focal parenchymal cord-like arrangement of cells	77 (77%)	151 (75.5%)	0 (0%)	0 (0%)	
	Diffuse parenchymal cord-like arrangement of cells	14 (14%)	27 (13.5%)	0 (0%)	0 (0%)	
	Hemorrhage	32 (32%)	64 (32%)	0 (0%)	0 (0%)	
	Haemorrhage + Hemosiderin like pigments	12 (12%)	21 (10.5%)	0 (0%)	0 (0%)	
	Edema	49 (49%)	95 (47.5%)	0 (0%)	0 (0%)	
	Edema + Necrosis	9 (9%)	16 (8%)	1 (5%)	2 (5%)	
Histological Changes in the Medulla	Cell size	Normal	Normal	Normal	-	-
	Extension of medulla	25 (25%)	44 (22%)	0 (0%)	-	
	Nuclear prominence	71 (71%)	139 (69.5%)	0 (0%)	-	
	Sinusoidal dilatation	99 (99%)	195 (97.5%)	0 (0%)	-	
	Hemorrhage	16 (16%)	30 (15%)	0 (0%)	-	
	Edema	19 (19%)	35 (17.5%)	0 (0%)	-	
	Necrosis	1 (1%)	2 (1%)	0 (0%)	-	

**Figure 7:**
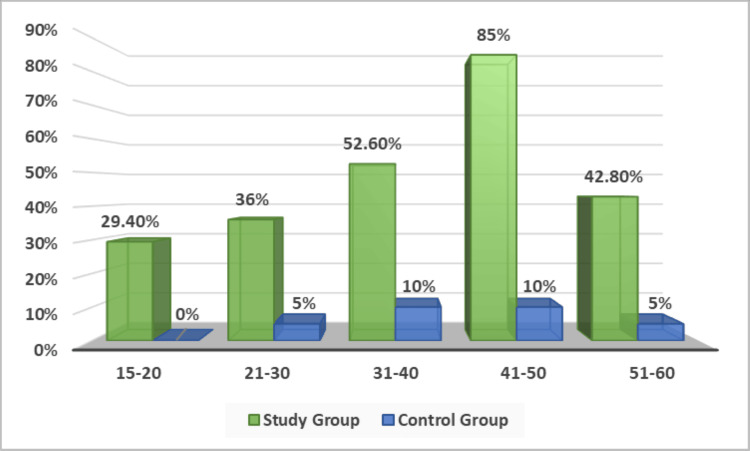
Age-wise distribution of the proportion of cases with nodular hyperplasia in the study and control groups.

Nodularity of the adrenal cortex was prevalent in the study group, with 48% showing focal nodularity. Control cases exhibited nodular hyperplasia in 4 out of 20 individuals. The presence of nodularity correlated with age at death, increasing in suicidal individuals up to 50 years of age. This difference was statistically significant (p=0.026) (Table 2). The presence of nodularity in the adrenal cortex was highly affected by age at the time of death; in suicidal individuals, adrenal cortical nodularity increased with increasing age up to 50 years of age. In the control group, among the age group of 15-20 years, none of the cases showed nodularity of the adrenal cortex (Figure 7).

The zona fasciculata was most affected in the study group, showing extensions, irregular thinning, prominent nuclei, dilated sinusoids, parenchymal cord-like arrangements, lipid depletion, and hemorrhage. These changes were unique to the suicidal cases and were not observed in the control group (Table 2, Figure 5). The zona reticularis was severely impacted in suicidal cases, with extensions, dark-staining nuclei, sinusoidal dilation, parenchymal tabulation, severe lipid depletion, hemorrhage, and edema. These changes were absent in control cases (Table 2, Figure 5). Some changes were common in both groups, though more frequent in suicidal cases, such as mild lipid depletion and necrosis with edema (Table 2). Specific medullary changes were noted in suicidal cases, including extension, nuclear prominence, sinusoidal dilation, hemorrhage, edema, and necrosis. These changes were not observed in control cases (Table 2, Figure 5).

There were scores recorded and given a scale score depending upon the change in the two groups, with a score of 1 given for every change. This has been depicted in Table 3.

**Table 3:** Comparison of scores in two groups. Scale score = for every change the score is 1.

Variables	Study Group						Control group					
	Capsular Hemorrhage	Nodular Hyperplasia	Zona Glomerulosa	Zona Fasciculata	Zona Reticularis	Medulla	Capsular Hemorrhage	Nodular Hyperplasia	Zona Glomerulosa	Zona Fasciculata	Zona Reticularis	Medulla
Extension	1	1	0	1	1	1	1	1	0	0	0	0
Cell size			Normal	Normal	Normal	Normal			0	Normal	Normal	0
Nuclear prominence			1	1	1	1			0	0	0	0
Sinusoidal dilatation			1	1	1	1			0	0	0	0
Focal lipid depletion			0	1	1	0			0	1	1	0
Diffuse lipid depletion			0	1	1	0			0	0	0	0
Focal parenchymal cord-like arrangement of cells			0	1	1	0			0	0	0	0
Diffuse parenchymal cord-like arrangement of cells			0	1	1	0			0	0	0	0
Hemorrhage			1	1	1	1			0	0	0	0
Hemorrhage + Hemosiderin-like pigments			1	1	1	0			0	0	0	0
Edema			1	1	1	1			0	1	0	0
Necrosis			0	1	1	1			0	0	0	0
Necrosis+Edema			0	1	1	1			0	0	1	0
Total	1	1	5	12	12	7	1	1	0	2	2	0
Grand Total	Case Score = 38						Control Score = 6					

## Discussion

The age and gender of the deceased did not exhibit a discernible impact on the histological appearance of the adrenal gland in both the study and control groups, except for the presence and severity of nodular hyperplasia. While many histological changes were shared between both glands, variations in severity were noticeable either between glands or within different regions of the same gland in a few instances. This suggested that both adrenal glands reacted differently to both acute and chronic stress. No specific histological changes were identified as exclusive to the cause or mode of death. Cell size remained consistent in both groups and thus was considered within the normal range. Hemorrhage and congestion were interpreted as reactive changes that manifested during the acute stress of dying, particularly in cases where survival occurred for some time before death. Consequently, it was challenging to differentiate between individuals exposed to the acute stress of dying and those experiencing chronic stress in the suicidal group. In the current study, capsular hemorrhage (Figure 1) was observed in 98% of cases and 40% of controls. A reddish appearance of the adrenal gland, consistent with previous findings by Patra S et al. [7, 8], was noted in 97% of suicidal cases and 15% of control cases.

Regarding nodularity (Figure 2), 48% of the suicidal group and 20% of the control group exhibited this feature. Adrenal nodular hyperplasia and nodule formation in the zona fasciculata were also documented by Braunstein H and Yamaguchi BT Jr. [9] in 92% of sudden deaths and 100% with chronic stress. Consequently, using nodular hyperplasia as a criterion for histological diagnosis of chronically stressed adrenal glands may be imprudent. Sasano H et al. [10] proposed an increased incidence of nodule formation with increasing age, aligning with the current study's findings, where it was more frequently observed in individuals aged 31-50 years and less common among those aged 15-20 years and over 50 years. This age-related trend was attributed to the heightened stress experienced by individuals in these age groups due to factors such as frustration, social, financial, marital issues, sudden emotional stimulation, and challenging circumstances. The failure to maintain homeostasis likely leads to localized hyperplasia/nodule formation as a coping mechanism for chronic stress.

Findings from Symington T et al. [11], Bornstein SR et al. [12], Patra S et al. [7, 8], and Sarkar A et al. [13] regarding the zonal extension of the adrenal cortex and irregularities (Figure 3) are considered specific to conditions of chronic stress. Bornstein SR et al. [12], Patra S et al. [7, 8], and Sarkar A et al. [13] specifically noted this change in suicidal cases, while Symington T et al. [11] observed it in burn patients who succumbed after prolonged hospitalization, exposed to chronic stress. Braunstein H and Yamaguchi BT Jr. [9] reported a more pronounced occurrence in those exposed to chronic stress. In this study, zonal extension, especially in the zona fasciculata, was found to be specific to chronic stress in suicidal cases. A slight extension of the zona reticularis was also observed, aligning with findings by Patra S et al. [7, 8], but contrary to Braunstein H and Yamaguchi BT Jr. [9], who depicted the zona reticularis as more regular and less variable in thickness. The medulla displayed marked variation in thickness and prominence in the present study, with a prominent extension observed in 25% of suicidal cases. Given the frequent poor preservation of the medulla postmortem, caution is warranted in drawing conclusive observations. Similar findings were reported by Braunstein H and Yamaguchi BT Jr. [9] in the deaths of individuals with chronic illness.

Consistent with Symington T et al. [11], Braunstein H and Yamaguchi BT Jr. [9], and Patra S et al. [7, 8], this study revealed marked irregular thinning of the zona glomerulosa with shrunken cells and increased nuclear density (Figure 4) in 88% of cases, a feature regarded as specific to chronic stress conditions and suicidal cases. Such thinning was absent in the control group. Undeniably, irregularity of the glomerulosa is a characteristic feature of human adrenal tissue. The thickness of the glomerulosa varied within the same section, ranging from a few small individual cells beneath the capsule to several multi-cellular globoid masses. There seemed to be a reciprocal relationship with the width of the zona fasciculata, suggesting that the thickness of the glomerulosa is influenced by the encroachment upon it by the extension of the zona fasciculata

Opinions from Sarason EL [14], Rogers WF Jr. and Williams RH [15], Zamcheck N [16], Ayers WW et al. [17], and Stoner HB et al. [18] suggest variations in intracellular lipids as a reactive change in the adrenal cortex under stressful conditions (Figure 5). Sayers G [19] noted that lipid depletion indicates adrenal gland hyperactivity, as seen in stress conditions. Symington T et al. [11], Braunstein H and Yamaguchi BT Jr. [9] observed focal lipid depletion in acute stress and diffuse/complete lipid depletion in chronic stress. Stoner HB et al. [18] observed a completely lipid-depleted adrenal in cases of chronic prolonged stress. Similarly, Willenberg HS et al. [20] and Patra S et al. [7] noted mild, moderate, and extensive grades of lipid depletion in suicidal cases, with no depletion observed in control cases. These findings align with the current study, which showed lipid depletion in all suicidal cases, with diffuse lipid depletion in 47% and focal depletion in 53% of cases. While 45% of cases exposed to the acute stress of dying exhibited focal depletion, none displayed diffuse lipid depletion. Therefore, the pattern of lipid depletion, along with other changes indicative of chronic stress, can serve as a significant criterion for distinguishing individuals dying suddenly from suicidal cases exposed to chronic stress. Focal loss of lipids in normal control individuals is likely attributed to the early response of the adrenal gland to acute stress.

The present study's observation, indicating focal areas of parenchymal cord-like arrangements of cells in 53% of cases and a diffuse change in the zona fasciculata in 46% of cases (Figure 4), appears to align with the findings of Patra S et al. [7], who specifically reported such arrangements of cells in suicidal cases. This specific finding holds significance in the context of suicidal death in individuals exposed to chronic stress. The proposed explanation is that, due to lipid depletion and cytological degenerative changes in adrenal cortical cells, the solid cords of cells in the outer parts of the cortex, corresponding to the position of the outer zona fasciculata, break up to form spaces. The cords of either clear or compact cells may be separated from the stroma by spaces, giving rise to a tubular appearance. The association between adrenal sinusoidal dilation (Figure 5) and chronic stress, noted in the present study, was also observed by Patra S et al. [8]. In both studies, 98-99% of suicidal cases exhibited dilated prominent sinusoids in the zona glomerulosa, fasciculata, reticularis, and adrenal medulla. This finding can be considered specific to the adrenal gland of individuals exposed to chronic stress, as it was absent in controls. This might be attributed to increased vascular demands in response to the heightened activity of the adrenal gland, adapting to the challenges posed by chronic stress.

Nuclear prominence (Figure 4) observed in the present study is in line with the findings of Symington T et al. [11], Worth CT et al. [21], Li KH et al. [22], and Patra S et al. [8]. In the current study, 95% of cases exhibited prominent nuclei in the zona fasciculata and reticularis, 68% in the glomerulosa, and 71% in the medulla. The suggested reason for the presence of prominent hyperchromatic nuclei is that cells of the adrenal gland exposed to chronic stress are actively dividing and rich in nucleic acids. RNA is abundant in compact gland cells, displaying focal and extreme lipid depletion, except where degenerative changes occur. The observation of adrenal hemorrhage and necrosis (Figure 5) associated with suicidal cases in the present study appears to be specific to the chronic stress condition. In this study, 7%, 8%, 32%, and 16% of cases showed hemorrhage in the zona glomerulosa, fasciculata, reticularis, and medulla, respectively, with none in controls. Symington T et al. [11] described similar changes in severe fatal burns, occurring as early as 24 hours after injury and persisting for up to 10 days. Hence, caution should be exercised in using these changes to differentiate chronically stressed adrenal glands from those exposed to the acute stress of dying.

Limitations

Along with these useful outcomes, there are a few potential limitations that could be addressed in future studies or that might have influenced the study results. Variability in histological interpretation can influence the results, as reporting of the findings seen on a microscope may vary among pathologists. Moreover, employing a larger sample size and categorizing it depending on the cause of death could provide a larger number of variables and study groups.

## Conclusions

In suicidal cases, a range of histological changes was observed, including capsular hemorrhage, nodular hyperplasia, and irregular thinning of the zona glomerulosa, with shrunken cells exhibiting increased nuclear density. Additionally, there was a zonal extension of the fasciculata, reticularis, and medullary zones, lipid depletion in focal and diffuse patterns, and parenchymal cord-like arrangements of cells in the fasciculata and reticularis layers. Sinusoidal dilatation, nuclear prominence, hemorrhage, hemosiderin-like pigments, edema, and necrosis were consistently observed across all zones. Contrastingly, in cases of acute stress, the only notable histological changes included focal lipid depletion in the zona fasciculata and reticularis, capsular hemorrhage, nodular hyperplasia, and hemorrhage and necrosis, along with edema. However, the proportion and severity of these changes were lower compared to the suicidal group. Consequently, these findings may be considered non-specific for differentiating between acute and chronic stress conditions.
